# Over-expression of DNA-PKcs in renal cell carcinoma regulates mTORC2 activation, HIF-2α expression and cell proliferation

**DOI:** 10.1038/srep29415

**Published:** 2016-07-14

**Authors:** Bing Zheng, Jia-Hui Mao, Xiao-Qing Li, Lin Qian, Hua Zhu, Dong-hua Gu, Xiao-dong Pan

**Affiliations:** 1 Department of Urology, The Second Affiliated Hospital of Nantong University, Nantong 226000, China; 2 Department of pathophysiology, Nantong University School of Medicine, Nantong, China

## Abstract

Here, we demonstrated that DNA-PKcs is over-expressed in multiple human renal cell carcinoma (RCC) tissues and in primary/established human RCCs. Pharmacological or genetic inhibition of DNA-PKcs suppressed proliferation of RCC cells. DNA-PKcs was in the complex of mTOR and SIN1, mediating mTORC2 activation and HIF-2α expression in RCC cells. Inhibiting or silencing DNA-PKcs suppressed AKT Ser-473 phosphorylation and HIF-2α expression. *In vivo*, DNA-PKcs knockdown or oral administration of the DNA-PKcs inhibitor NU-7441 inhibited AKT Ser-473 phosphorylation, HIF-2α expression and 786-0 RCC xenograft growth in nude mice. We showed that miRNA-101 level was decreased in RCC tissues/cells, which could be responsible for DNA-PKcs overexpression and DNA-PKcs mediated oncogenic actions in RCC cells. We show that DNA-PKcs over-expression regulates mTORC2-AKT activation, HIF-2α expression and RCC cell proliferation.

Renal cell carcinoma (RCC) is the most common renal malignancy. Its incidence is steadily rising around the world^1^,^2^,^3^,^4^. The majority of RCC patients are diagnosed at the advanced stages with local or systematic metastasis, and the prognosis of these patients is often poor^1^,^2^,^3^,^5^,^6^. Surgery treatment remains the only curable therapeutic option for RCC^1^,^7^,^8^,^9^. Chemotherapy, hormonal therapy or radiation therapy have only displayed limited values, probably due to pre-existing or acquired chemo-resistance from RCC cells^1^,^7^,^8^,^9^. Therefore, research groups are searching for valuable targets (*i.e.* novel oncogenes) for RCC, and developing possible intervention strategies^7^,^8^.

Recent studies have implicated a potential role of DNA-dependent protein kinase (DNA-PK) in cancer progression^10^,^11^,^12^,^13^. DNA-PK is a three protein complex comprising the catalytic subunit (DNA-PKcs) and Ku hetero-dimer (Ku-70/Ku-80)^14^,^15^. The catalytic subunit, DNA-PKcs, is a member of phosphatidylinositol 3-kinase (PI3K)-like protein kinases (PIKK)^14^,^15^. When activated, *i.e.* by irradiation, this 460-kDa serine/threonine protein kinase regulates non-homologous end joining (NHEJ) signaling to repair DNA double-strand breaks^14^,^15^. Studies have explored the potential role of DNA-PKcs in multiple tumors, and showed that DNA-PKcs could regulate AKT-mammalian target of rapamycin (mTOR) activation, thus regulating in cancer survival, proliferation and resistance to radiation/chemotherapy^12^,^13^,^16^,^17^,^18^. However, the expression and potential functions of DNA-PKcs in RCC are largely unknown.

In the current study, we show that DNA-PKcs is over-expressed in multiple human RCC tissues and cells, regulating mTOR complex 2 (mTORC2)-AKT activation, hypoxia-inducible factor-2α (HIF-2α) expression and RCC cell progression. Decreased level of miRNA-101 (miR-101), the anti-DNA-PKcs miRNA^19^, could be the reason of DNA-PKcs upregulation in RCC cells.

## Results

### DNA-PKcs over-expression in human RCC cells and tissues

**Figure 1:**
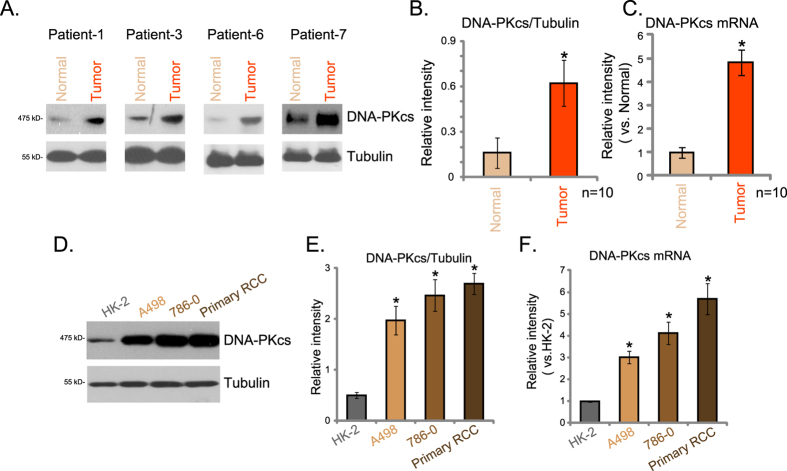
DNA-PKcs over-expression in human RCC cells and tissues. DNA-PKcs protein (**A,B**) and mRNA (**C**) expressions in human RCC tissues (“Tumor”) and surrounding normal renal tissues (“Normal”) were shown. DNA-PKcs protein (**D,E**) and mRNA (**F**) expressions in A498, 786-0 and primary human RCC cells as well as in HK-2 cells were shown. *p < 0.05 vs. “Normal” tissues (**B,C**). *p < 0.05 vs. “HK-2” (**D,E**).

First, we examined the expression of DNA-PKcs in human RCC tissues. As shown in Fig. 1A, compared to the surrounding normal renal tissues, DNA-PKcs protein expression level was significantly higher in RCC tissues. We analyzed a total of ten independent RCC tissues, DNA-PKcs expression in RCC tissues was about 4-times higher than that in normal renal tissues (Fig. 1B). Real-time PCR assay results showed that DNA-PKcs mRNA level was also increased in RCC tissues (Fig. 1C).

Expression of DNA-PKcs in human RCC cells was also analyzed. As shown in Fig. 1D,E, DNA-PKcs protein expression was significantly higher in established (A498 and 786-0 lines)^20^ and primary human RCC cells than that in non-cancerous proximal tubule epithelial HK-2 cells^20^,^21^. In addition, DNA-PKcs mRNA level was over-expressed in above HCC cells (Fig. 1F). Thus, these results show that DNA-PKcs is over-expressed in human RCC tissues and RCC cells.

### DNA-PKcs inhibitors induce proliferation inhibition and apoptosis in RCC cells

**Figure 2:**
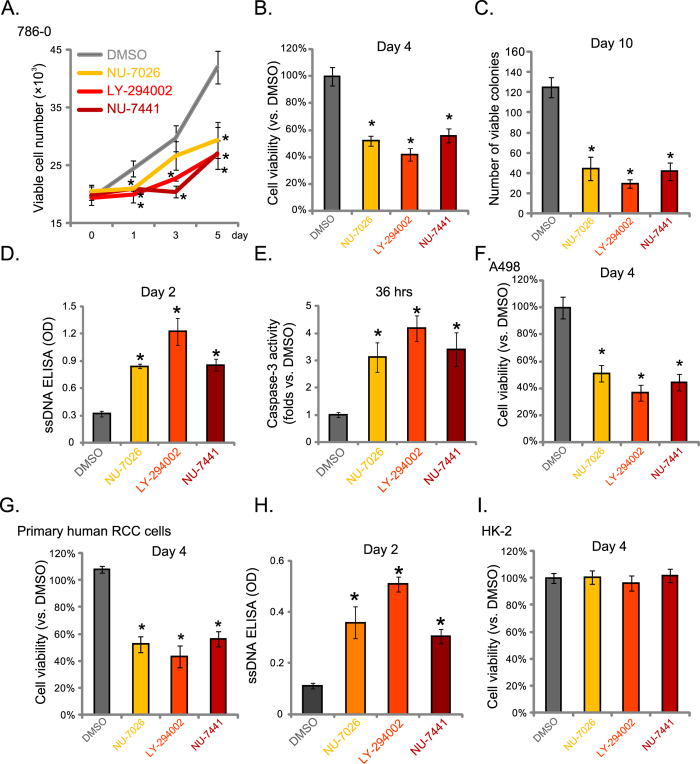
DNA-PKcs inhibitors induce proliferation inhibition and apoptosis in RCC cells. 786-0 (**A**–**E**), A498 (**F**) or primary human RCC cells (**G**,**H**) as well as the HK-2 human proximal tubule epithelial cells (**I**) were treated with NU-7026 (5 μM), NU-7441 (5 μM), LY-294002 (1 μM) or vehicle control (0.1% DMSO) for applied time, cell proliferation was analyzed by viable cell counting assay (**A**, for 786-0 cells), MTT assay (**B**,**F**,**G**,**I**) or clonogenicity assay (**C**, for 786-0 cells); Cell apoptosis was tested by the ssDNA ELISA assay (**D**,**H**) or the caspase-3 activity assay (**E**, for 786-0 cells). Experiments in this figure were repeated three times, and similar results were obtained. For each assay, n = 5. *p < 0.05 vs. “DMSO” group.

Above results demonstrate that DNA-PKcs is over-expressed in human RCC cells and RCC tissues. Next, we studied the potential effect of DNA-PKcs in RCC cell proliferation. Three different DNA-PKcs inhibitors, including NU-7026^22^, NU-7441^23^ and LY-294002^24^ were applied. Simply through the viable cell (trypan blue exclusive) counting assay, our results showed that the DNA-PKcs inhibitors remarkably inhibited 786-0 RCC cell proliferation (Fig. 2A). Meanwhile, the results of MTT viability assay (Fig. 2B) and clonogenicity assay (Fig. 2C) further confirmed the anti-proliferative activity by these DNA-PKcs inhibitors. We also noticed significant apoptosis activation in 786-0 cells after treatment of DNA-PKcs inhibitors, which was shown by ssDNA apoptosis ELISA assay (Fig. 2D) and caspase-3 activity assay (Fig. 2E).

These DNA-PKcs inhibitors were also anti-proliferative in A498 RCC cells (Fig. 2F), and in primary human RCC cells^20^ (Fig. 2G). Apoptosis induction, evidenced by ssDNA ELISA OD increase (Fig. 2H), was also observed in the primary cancer cells after the DNA-PKcs inhibitor treatment. On the other hand, the proliferation of non-cancerous HK-2 cells (low DNA-PKcs expression, Fig. 1)^21^ were not affected by the same DNA-PKcs inhibitor treatment (Fig. 2I). Note that expression of DNA-PKcs was not affected by these inhibitors in above cells (Data not shown). Together, these results demonstrate that DNA-PKcs inhibitors exert anti-proliferative and pro-apoptotic activities to cultured RCC cells.

### DNA-PKcs knockdown inhibits RCC cell proliferation

**Figure 3:**
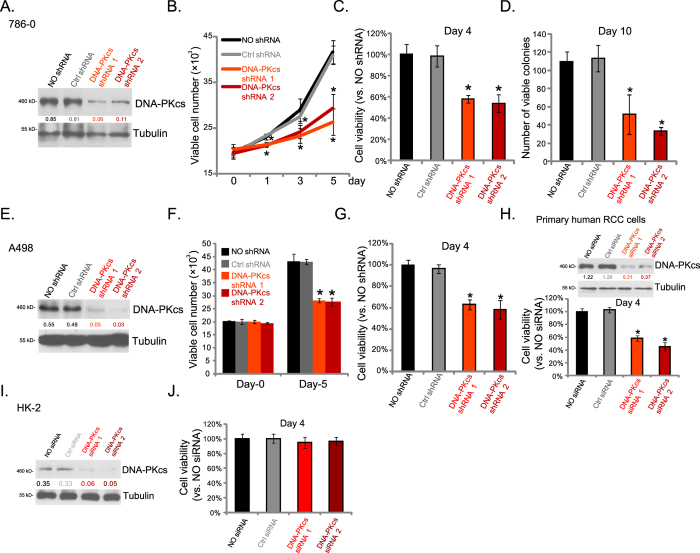
DNA-PKcs knockdown inhibits RCC cell proliferation. Stable 786-0 (**A–D**) or A498 (**E–G**) RCC cells, expressing DNA-PKcs shRNA (-1/-2), scramble control shRNA (“Ctrl shRNA”) or without shRNA (“NO shRNA”), were cultured in FBS-containing medium for applied time, expression of DNA-PKcs and β-tubulin (**A,E**) was shown, cell proliferation was tested by viable cell counting (**B,F**), MTT assay (**C,G**) or clonogenicity assay (**D**, for 786-0 cells). Primary human RCC cells (**H**) or HK-2 human proximal tubule epithelial cells (**I,J**), transfected with scramble control shRNA (“Ctrl siRNA”), or DNA-PKcs siRNA (-1/-2), were cultured in FBS-containing medium for applied time, DNA-PKcs and tubulin protein expressions were shown (**H**, upper panel and **I**), cell proliferation was tested by MTT assay (**H**, lower panel and **J**). DNA-PKcs protein expression (vs. tubulin) was quantified (**A,E,H,I**). Experiments in this figure were repeated three times, and similar results were obtained. *p < 0.05 vs. “Ctrl shRNA/siRNA” group.

Above evidences indicate that DNA-PKcs is important for RCC cell proliferation. To further confirm our hypothesis, siRNA/shRNA strategy was applied to selectively knockdown DNA-PKcs in RCC cells. Stable DNA-PKcs-knockdown 786-0 cells were established through shRNA lentiviral infection (See methods). Western blot results in (Fig. 3A) demonstrated that DNA-PKcs expression was significantly downregulated in stable cells expressing DNA-PKcs shRNAs (-1/-2). As a consequence, the 786-0 cell proliferation, tested by viable cell counting assay (Fig. 3B), MTT viability assay (Fig. 3C) and clonogenicity assay (Fig. 3D), was remarkably inhibited in DNA-PKcs-silenced cells (Fig. 3B–D). Similar results were also observed in A489 RCC cells (Fig. 3E–G). Scramble control shRNA (“Ctrl shRNA”) had no effect on RCC cell proliferation (Fig. 3A–G).

For the primary human RCC cells, siRNA method was applied to transiently knockdown DNA-PKcs, and Western blot assay showed DNA-PKcs silence by the two targeted siRNAs (Fig. 3H, upper). Primary human RCC cell proliferation, tested by MTT assay, was also inhibited by the non-overlapping DNA-PKcs siRNAs (Fig. 3H, lower). The proliferation of proximal tubule epithelial HK-2 cells was not affected by DNA-PKcs siRNAs (Fig. 3I,J). Thus, these results show that DNA-PKcs knockdown inhibits RCC cell proliferation *in vitro*.

### DNA-PKcs is in the complex of mTOR and SIN1, required for mTORC2 activation and HIF-2α expression

**Figure 4:**
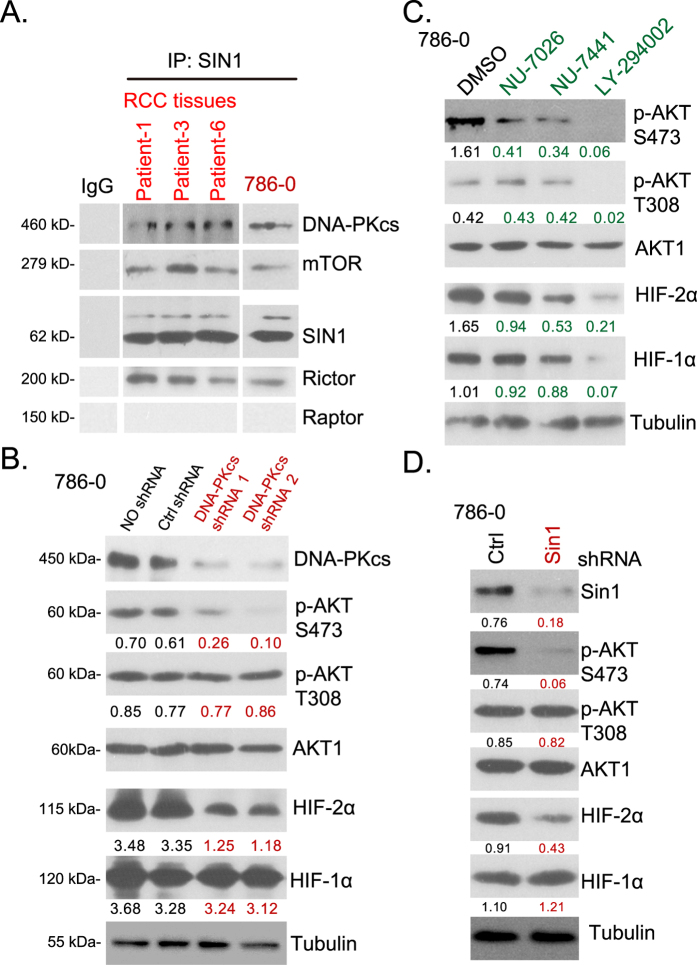
DNA-PKcs is in the complex of mTORC2, regulating AKT Ser-473 phosphorylation and HIF-2α expression. The association between DNA-PKcs, mTOR, Rictor and SIN1 (but not Raptor) in human RCC tissue lysates or 786-0 lysates was tested by Co-IP assay (**A**). Expression of listed proteins in 786-0 cells expressing DNA-PKcs shRNA (-1/-2) (**B**), SIN1 shRNA (**D**) or scramble control shRNA (“Ctrl shRNA”) was shown. 786-0 cells were treated with NU-7026 (5 μM), NU-7441 (5 μM), LY-294002 (1 μM) or vehicle control (0.1% DMSO) for 12 hours, expression of listed proteins was tested by Western blots (**C**). AKT phosphorylation (vs. total AKT) and HIF-1/2α expression (vs. Tubulin) were quantified. Experiments in this figure were repeated three times, and similar results were obtained.

Recent studies have demonstrated a potential role of DNA-PKcs in mTORC2 activation^16^,^25^,^26^. It has been shown that DNA-PKcs could form a complex with SIN1, a key component of mTORC2^27^,^28^, thus regulating AKT Ser-473 phosphorylation^16^,^25^. Thus, we examined the potential role of DNA-PKcs in AKT-mTOR signaling activation in RCC tissues. First, using Co-IP assay, we noticed a physical interaction between DNA-PKcs, SIN1, Rictor and mTOR in multiple human RCC tissues, and in 786-0 RCC cells (Fig. 4A). Raptor, the mTORC1 component^29^, was not in the complex (Fig. 4A). Same IP method failed to detect a significant SIN1-DNA-PKcs association in the above normal renal tissues (Data not shown), possibly due to low expression of both proteins (Fig. 1). As shown in Fig. 4B, DNA-PKcs-shRNA knockdown significantly inhibited AKT Ser-473 phosphorylation in 786-0 cells. Yet AKT Thr-308 phosphorylation was almost unaffected. Meanwhile, expression of HIF-2α, a mTORC2-regulated gene^30^, was also downregulated with DNA-PKcs knockdown (Fig. 4B). The HIF-1α expression, which was mainly regulated by mTORC1^30^, was not changed (Fig. 4B).

In addition, DNA-PKcs inhibitors, including NU-7026, NU-7441 and LY-294002, dramatically inhibited AKT Ser-473 phosphorylation and HIF-2α expression in 786-0 cells (Fig. 4C). Since LY-294002 was also a PI3K-AKT-mTOR pan inhibitor^31^, it thus blocked AKT Thr-308 phosphorylation and downregulated HIF-1α expression in 786-0 cells (Fig. 4C). As expected, SIN1-shRNA expressing cells showed similar results as DNA-PKcs-shRNA cells, showing decreased AKT Ser-473 phosphorylation and HIF-2α expression (Fig. 4D). AKT Thr-308 phosphorylation and HIF-1α expression were again not affected by SIN1 shRNA knockdown (Fig. 4D). Note that above experiments were also repeated in A498 cells and primary human HCC cells, and similar results were obtained (Data not shown). Together, these results indicate that DNA-PKcs is in the complex of mTORC2, regulating AKT Ser-473 phosphorylation and HIF-2α expression in RCC cells.

### DNA-PKcs inhibition or silence suppresses AKT Ser-473 phosphorylation, HIF-2α expression and 786-0 xenograft growth *in vivo*

**Figure 5:**
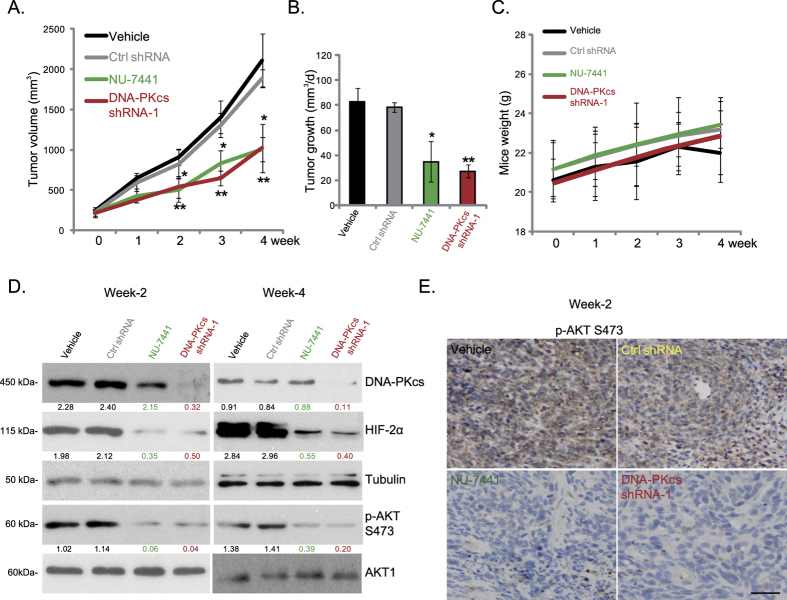
DNA-PKcs inhibition or knockdown inhibits 786-0 xenograft growth *in vivo*. Xenograft growth of 786-0 cells expressing scramble control shRNA (“Ctrl shRNA”), DNA-PKcs shRNA(-1) or NO-shRNA in nude mice, treated with vehicle or NU-7441 (10 mg/kg daily) was shown. Each treatment group comprised 10 mice. Mean estimated tumor volume (**A,B**) and mice body weight (**C**) were presented. Week-2 and week-4 after initial treatment, 786-0 xenografts were isolated from experimental mice (one mice per group), AKT Ser-473 phosphorylation, HIF-2α expression and equal loadings were analyzed by Western blots (**D**) or IHC staining (**E**, for p-AKT Ser-473). DNA-PKcs/HIF-2α expression (vs. Tubulin) and AKT Ser-473 phosphorylation (vs. total AKT) were quantified (**D**). Bar = 50 μm (**E**). Experiments in this figure were repeated twice, and similar results were obtained. *p < 0.05 vs. “vehicle” group. **p < 0.05 vs. “Ctrl shRNA” group.

We tested the potential role of DNA-PKcs in RCC cell growth *in vivo*. Results demonstrated that oral administration of a single dose of the DNA-PKcs inhibitor NU-7441 (10 mg/kg, daily for three weeks) resulted in a significant inhibition of 786-0 xenograft growth in nude mice (Fig. 5A). Meanwhile, the *in vivo* growth of stable 786-0 cells with DNA-PKcs shRNA was also slower than the cells expressing scramble control shRNA (Fig. 5A). The daily xenograft growth volume was significantly lower with NU-7441 administration or DNA-PKcs silencing (Fig. 5B). Note that mice body weights were not affected by NU-7441 treatment nor by DNA-PKcs silencing (Fig. 5C). We also did not notice any signs of apparent toxicities, such as diarrhea, fever, severe piloerection or a sudden weight loss (>10%), in the tested animals (Data not shown).

The signaling changes in the above xenografted tumors were also analyzed. In line with the *in vitro* findings, Western blot analysis of 786-0 xenografts (week-2 and week-4 after initial treatment) showed that AKT Ser-473 phosphorylation and HIF-2α expression were both inhibited by NU-7441 administration or DNA-PKcs shRNA knockdown *in vivo* (Fig. 5D). DNA-PKcs band confirmed its silence in 786-0 xenografts expressing DNA-PKcs-shRNA-1 (last for at least 2 to 4 weeks, Fig. 5D). Immunohistochemistry (IHC) results further confirmed that NU-7441 administration or DNA-PKcs shRNA suppressed AKT Ser-473 phosphorylation in 786-0 xenografts (two weeks after initial treatment, Fig. 5E). Collectively, these results show that DNA-PKcs inhibition or silencing suppresses AKT Ser-473 phosphorylation, HIF-2α expression and 786-0 xenograft growth *in vivo*.

### miR-101 downregulation correlates with DNA-PKcs overexpression in RCC

**Figure 6:**
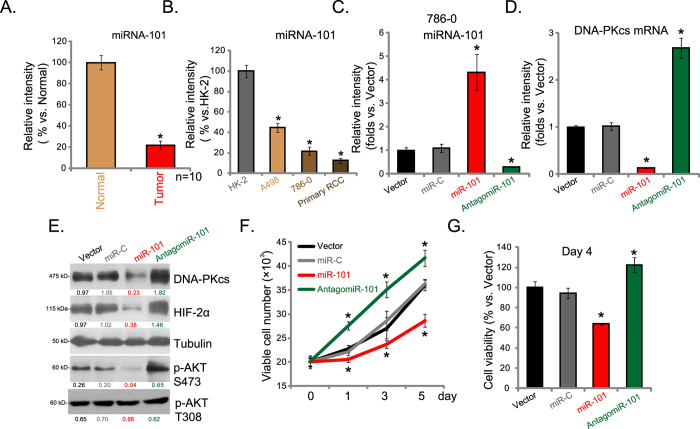
miR-101 downregulation correlates with DNA-PKcs overexpression in RCC. Relative miRNA-101 (miR-101) expression in human RCC tissues (“Tumor”) and surrounding normal renal tissues (“Normal”) as well as in established (A498 and 786-0)/primary human RCC cells or in HK-2 cells was shown (**A,B**). Stable 786-0 cells expressing miR-101, antagomiR-101, miRNA control (“miR-C”) or vector control (pSuper-puro, “Vector”) were subjected to real-time PCR assay, relative miR-101 expression and DNA-PKcs mRNA expression was shown (**C,D**). Expression of listed proteins in above cells was also shown (**E**), DNA-PKcs/HIF-2α expression (vs. Tubulin) and AKT Ser-473/Thr-308 phosphorylation (vs. Tubulin) were quantified (**E**). Above cells were also subjected to proliferation assay (**F**) and cell viability assay (**G**). Experiments in this figure were repeated three times, and similar results were obtained. *p < 0.05 vs. “Normal” group or HK-2 cell group (**A,B**). *p < 0.05 vs. “miR-C” group (**C,D,F,G**).

Above results have shown that DNA-PKcs overexpression regulates mTORC2 activation, HIF-2α expression and RCC cell proliferation. Next, we studied the underlying mechanisms of DNA-PKcs overexpression by focusing on miRNA regulation. A recent study by Yan *et al.* showed that miR-101 targeted 3′UTR of DNA-PKcs mRNA, leading to DNA-PKcs mRNA degradation^19^. We thus analyzed level of miR-101 in human RCC tissues and RCC cells. Real-time PCR assay results in (Fig. 6A) showed that miR-101 was significantly decreased in RCC tissues (“Tumor”) than that in surrounding normal renal tissues (“Normal”) (n = 10). In addition, miR-101 level was also lower in established (A498 and 786-0 lines) and primary human RCC cells, as compared to HK-2 cells (Fig. 6B).

To study whether low level of miR-101 is responsible of DNA-PKcs overexpression in RCC cells, we exogenously expressed miR-101 into 786-0 cells^32^. Real-time PCR assay results confirmed miR-101 over-expression in stable 786-0 cells with the miR-101 construct^32^ (Fig. 6C). As a result, DNA-PKcs mRNA (Fig. 6D) and protein (Fig. 6E) expression were both downregulated. On the other hand, introduction of antagomiR-101^32^ expectably downregulated miR-101 in 786-0 cells (Fig. 6C), leading to even higher DNA-PKcs expression (Fig. 6D,E). Importantly, in 786-0 cells, AKT Ser-473 phosphorylation (Fig. 6E), HIF-2α expression (Fig. 6E) as well as cell proliferation (Fig. 6F,G) were significantly inhibited by miR-101 over-expression, but were further potentiated with introduction of antagomiR-101 (Fig. 6E–G). Note that AKT Thr-308 phosphorylation was again not affected by miR-101 nor antagomiR-101 (Fig. 6E). Same experiments were also repeated in A498 cells, and similar results were obtained. These results suggest that miR-101 downregulation could be the key reason of DNA-PKcs overexpression and DNA-PKcs mediated oncogenic behaviors in RCC cells.

## Discussions

In the present study, our results indicate that DNA-PKcs might be a novel oncogene for the RCC. First, we demonstrate that DNA-PKcs is over-expressed in multiple human RCC tissues. Second, inhibition of DNA-PKcs, through pharmacological inhibitors or siRNA/shRNA knockdown, significantly reduced RCC cell proliferation *in vitro* and *in vivo*. Third, DNA-PKcs was found in the complex of mTORC2, and was required for AKT activation (Ser-473 phosphorylation) and HIF-2α expression in RCC cells. Thus, DNA-PKcs might be a valuable target for RCC intervention.

Overactivity of AKT is observed in many RCCs, which plays a vital role in cell survival, proliferation, migration, apoptosis-resistances and other cancerous behaviors^33^,^34^. Complete activation of AKT requires both Ser-473 and Thr-308 phosphorylations^33^,^34^. Studies have indicated a potential role of DNA-PKcs in regulating AKT activation. For example, Feng *et al.* showed that DNA-PKcs directly associates and activates AKT in the plasma membrane, causing a 10-fold enhancement of AKT activity^35^. Dragoi *et al.* demonstrated that DNA-PKcs co-localizes and activates AKT after CpG-DNA stimulation^36^. Recent studies have shed lights on how DNA-PKcs activates AKT. Tu *et al.* showed that Ultra Violet (UV) radiation leads to nuclear DNA-PKcs translocation to cytosol, where it forms a complex with mTORC2 component SIN1 to phosphorylate AKT at Ser-473^16^. Similarly, Xu and co-authors found that, upon low-dose X-ray irradiation (LDI), DNA-PKcs associates with mTORC2 to mediate AKT Ser 473 phosphorylation^25^. In the current study, we showed that DNA-PKcs formed a complex with mTOR and SIN1 in both human RCC tissues and RCC cells, and was required for mTORC2 activation (AKT Ser-473 phosphorylation) and HIF-2α expression. Inhibition or silencing of DNA-PKcs in RCC cells reduced AKT Ser-473 phosphorylation and HIF-2α expression. Thus, DNA-PKcs may regulate RCC cell proliferation through regulating mTORC2 signaling.

Studies have identified a strong association between pVHL (von Hippel–Lindau protein) mutation and RCC progression^37^,^38^. As an E3 ubiquitin ligase, pVHL promotes HIF-1α/2α degradation^38^. pVHL silencing or inactivation will thus cause HIF-1α/2α accumulation, leading to vascular endothelial growth factor (VEGF) production and tumor angiogenesis^38^. Studies have shown that 50% or more sporadic RCCs have somatic mutations in pVHL^38^. Although the role HIF-1α in tumor progression and angiogenesis has been extensively studied, existing evidences indicated that HIF-2α is far more important than HIF-1α in the pathogenesis of RCC^20^,^39^,^40^. As a matter of fact, HIF-2α silencing was shown to inhibit the ability of pVHL-knockout RCC cells to form tumors *in vivo*^39^. Kondo and colleagues showed that pVHL-mediated tumor suppression is abolished with overexpression of HIF-2α, but not HIF-1α^40^.

In the current study, we showed that HIF-2α expression was inhibited with DNA-PKcs silencing or blockage. These results were not surprising, since translation of HIF-2α is solely dependent upon the activity of mTORC2^30^, and we showed that DNA-PKcs was required for mTORC2 activation in RCC cells. As a matter of fact, SIN1 shRNA knockdown similarly decreased HIF-2α expression in 786-0 cells. Notably, HIF-1α expression was not affected by DNA-PKcs or SIN1 knockdown. One reason could be that HIF-1α translation is controlled mainly by mTORC1^30^. Collectively, we suggest that DNA-PKcs is in the complex of mTORC2, regulating AKT Ser-473 phosphorylation and HIF-2α expression in RCC cells.

miRNA-mediated gene regulation plays a fundamental role in controlling gene expression at the post-transcriptional level^41^. These miRNAs are vital in modifying many key biologic processes of human cells, possibly via regulating expression of signaling molecules including growth factors, cytokines, transcription factors and other proteins (*i.e.* DNA-PKcs^19^)^41^,^42^. In addition, recent studies have shown that at least half of the miRNAs are linked to human cancers, these miRNAs are either upregulated or downregulated in human cancer cells. Specifically, many oncogenes and tumor suppressor genes are virtually regulated by miRNAs^42^. DNA-PKcs is shown to be negatively regulated by miRNA-101^19^. In the current study, we showed that miR-101 level was significantly lower in human RCC tissues, and in established or primary RCC cells, which might be a reason for DNA-PKcs over-expression. Introduction of miR-101 in RCC cells downregulated DNA-PKcs expression, and inhibited AKT activation, HIF-2α expression and cell proliferation. Reversely, over-expression of antagomiR-101 downregulated miR-101, and further enhanced DNA-PKcs expression and RCC cell proliferation. These results indicate that miR-101 downregulation might be at least one key reason for DNA-PKcs overexpression in RCC cells.

In summary, our results demonstrate that DNA-PKcs over-expression in RCC cells regulates mTORC2-AKT activation, HIF-2α expression and RCC cell progression. DNA-PKcs might be a valuable target for RCC treatment.

## Methods

### Chemicals and antibodies

NU-7026 and NU-7441 were purchased from Calbiochem (San Diego, CA). LY294002 was purchased from Sigma Chemical Co. (St. Louis, Mo.). Antibodies of p-AKT (Ser 473, #9271), p-AKT (Thr 308, #9275), AKT (9272), HIF-1α (#3716), HIF-2α (#7096), and β-tubulin (#2128), mTOR (#2983), Raptor (#2280) and Rictor (#2114) were obtained from Cell Signaling Tech. Anti-mSIN1 Antibody (07-2276) was obtained from EMD Millipore (Shanghai, China).

### Cell culture

As previously reported^20^, established human RCC cell lines (786-0 and A498) were obtained from Shanghai Biological Institute (Shanghai, China). Cells were cultured in DMEM plus 10% fetal bovine serum (FBS, Gibco, Shanghai, China). HK-2 cells, an immortalized human proximal tubule epithelial cell line, were maintained in DMEM medium with necessary supplements^20^. For all the cell lines, DNA fingerprinting and profiling were performed every 6 months to confirm the origin of the cell line, and to distinguish the cell line from cross-contamination. All cell lines were subjected to mycoplasma and microbial contamination examination. Population doubling time, colony forming efficiency, and morphology under phase contrast were also measured every 6 months under defined conditions to confirm the phenotype of cell line.

### Human RCC tissues

Tissue specimens were obtained from ten RCC patients with total nephroureterectomy. All patients were administrated in the Second Affiliated Hospital of Nantong University. Each patient received no irradiation or chemotherapy prior to surgery. In each fresh-isolated specimen, tumor tissue and the surrounding normal renal tissue were separated and paired. Tissues were thoroughly washed in PBS with antibiotics and DTT (2.5 mM, Sigma), and then minced into small pieces, which were then maintained in DMEM plus 10% FBS and necessary antibiotics. Tissues were lysed and analyzed by Western blots and real-time PCR. All patients enrolled provided individual written-informed consent. Using human specimens in this study was approved by the Nantong University’s Scientific Ethical Committee (Approve ID: 2013-002). The methods were carried out in accordance with the principles set out in the Declaration of Helsinki and the NIH Belmont Report.

### Primary culture of human RCC cells

Part of the minced RCC tumor tissues were also subjected to collagenase I (Sigma, 0.05% w/v) digestion for 30 min. Afterwards, individual cells were pelleted and rinsed twice with DMEM, and then cultured in DMEM, supplied with 10% FBS, 2 mM glutamine, 1 mM pyruvate, 10 mM HEPES, 100 units/mL penicillin/streptomycin, 0.1 mg/mL gentamicin, and 2 g/liter fungizone. Primary RCC cells of passage 3–6 were utilized for experiments.

### Real-time PCR

Total RNA was extracted through the SV total RNA isolation kit (Promega), and reverse transcription was performed through the TOYOBO ReverTra Ace-a RT-PCR kit (TOYOBO, Japan). We mixed the cDNA with SYBR Green PCR Master Mix and analyzed it by real-time PCR using the ABI7700 (Applied Biosystems). The primer sequences were as follows: DNA-PKcs primers, forward 5′-CCAAGTCCAACACCAAGTAGCCACCCA-3′; and reverse 5′-CCGCCATGCCGCCGAGTCCC-3′^43^. Glyceraldehyde-3-phosphate dehydrogenase (GAPDH) primers, forward, 5′-GAAGGTGAAGGTCGGAGTC-3′; reverse, 5′-GAAGATGGTGATGGGATTTC-3′; MiR-101: forward: 5′-CGG CGG TAC AGT ACT GTG ATA A-3′, reverse: 5′-CTG GTG TCG TGG AGT CGG CAA TTC-3′ (Universal stem-loop primer)^32^,^44^. After amplification, melt curve analysis was performed to calculate product melting temperature. For normalization, GAPDH was tested as the reference gene, and ^ΔΔ^Ct method was applied.

### Single-stranded DNA analysis of apoptosis

The single-stranded DNA (ssDNA) Apoptosis ELISA Kit (Chemicon International, Temecula, CA) was utilized to quantify cell apoptosis. This assay was based on selective DNA denaturation in apoptotic cells by formamide, and detection of the denatured DNA by monoclonal antibody to single-stranded DNA. The detailed procedure was described in other studies^45^,^46^.

### Caspase-3 activity assay

Caspase-3 activity assay was described in our previous study^20^. Briefly, ten micrograms of cytosolic extracts per treatment were added to caspase assay buffer (312.5 mM HEPES, pH 7.5, 31.25% sucrose, 0.3125% CHAPS) and the caspase-3 substrate (Calbiochem, Darmstadt, Germany). The release of 7-amido-4-(trifluoromethyl)-coumarin (AFC) was quantified via a Fluoroskan system set to an excitation value of 355 nm and emission value of 525 nm.

### Western blots

As previously reported^20^, cells or minced tissues were lysed by the lysis buffer containing 10 mM Tris–HCl (pH 7.4), 200 mM NaCl, 1 mM EGTA, 1% Triton X-100, 1 mM phenylmethylsulfonylfluoride, 10 μg/mL leupeptin, 10 μg/mL aprotinin, 100 mM NaF and 200 μM sodium orthovanadate. Aliquots of 30 μg of protein samples were separated by electrophoresis in SDS-PAGE, transferred to the PVDF membrane and detected with the specific antibody. The immunoreactive proteins after incubation with appropriately labeled secondary antibody were detected with an enhanced chemiluminescence (ECL) detection kit (Amersham, Buckinghamshire, UK). Band intensity was quantified by ImageJ software (NIH) after normalization to the loading control.

### Co-Immunoprecipitation (Co-IP)

As previously reported^20^, aliquots of 1000 μg of protein samples in 1 mL of lysis buffer from each treatment were pre-cleared by incubation with 30 μL of protein A/G Sepharose (Sigma) for 2 hours at 4 °C rotation. The pre-cleared samples were incubated with the specific anti-SIN1 antibody (1 μg/mL) overnight at 4 °C rotation. 20–30 μL of protein A/G Sepharose were added to the samples 2 hours at 4 °C rotation. The beads were washed and boiled, followed by Western blot assay.

### MTT assay of cell proliferation

Cells were seeded onto 96-well plates (3,000 per well) and allowed to attach overnight. After treatment of cells, cell viability/proliferation was tested using MTT [3-(4,5-dimethylthiazol-2-yl)-2,5-diphenyl tetrazolium bromide] assay (Sigma) according to the manufacturer’s instructions. Absorbance was measured at 490 nm through a Microplate Reader. The OD value of treatment group was always normalized to that of the control group.

### Clonogenic assay

786-0 cells were plated onto 60 mm plates with 2000 cells per plate. After treatment of cells, surviving colonies were fixed, stained with coomassie blue, and manually counted.

### shRNA and stable cell selection

For shRNA experiments, lentiviral particles were produced by constructing a lentiviral GV248 expression vector (Genechem, Shanghai, China) containing a puromycin resistance gene and either scramble control shRNA (5′-AATTCTCCGAACGTGTCACGT)^47^, shRNAs to DNA-PKcs (5′-GAACACTTGTACCAGTGTT, DNA-PKcs shRNA-1)^47^ and (5′-GATCGCACCTTACTCTGTT, DNA-PKcs shRNA-2)^48^. SIN1 shRNA lentiviral particles were purchased from Santa Cruz Biotech (Santa Cruz, CA). For infection, RCC cells were grown in 6-well culture plates in the presence of 2.0 μg/mL polybrene (Sigma) to 60% confluence, lentiviral-shRNAs were added to the cells. Virus-containing medium was replaced with fresh medium after 12 hours. Stable clones were selected by puromycin (0.5 μg/mL) for 10 days, expression of targeted protein in the resistant colonies was tested by Western blots or real-time PCR.

### siRNA knockdown of DNA-PKcs

To transiently knockdown DNA-PKcs in primary human RCC cells, siRNA method was utilized. SiRNA sequences for human DNA-PKcs were 5′-GAUCGCACCUUACUCUGUUdTdT-3′, (DNA-PKcs siRNA-1)^49^ and 5′-AGGGCCAAGCUGUCACUCU-3′ (DNA-PKcs siRNA-2)^49^, and were synthesized by Genechem (Shanghai, China). A negative control scramble siRNA was purchased Genechem. SiRNA (200 nM each) transfection was performed through Lipofectamine 2000 (Invitrogen, Carlsbad, CA) according to the standard procedure. The transfection took 48 hours.

### miRNA constructs and transfection

miRNA-101 (miR-101) expression pSuper-puro-GFP vector and antagomiR-101 expression vector as well as miR-control (“miR-C”) and pSuper-puro-GFP vector were gifts from Dr. Lu’s Lab at Nanjing Medical University^32^. Cells were seeded onto 6-well plates at 50% confluence with 2.0 μg/mL polybrene (Sigma). After 24 hours, cells were transfected using Lipofectamine 2000 transfection reagent (Invitrogen, USA). Twelve hours later, transfection medium was replaced with 2 mL of complete medium. Puromycin (2.5 μg/mL, Sigma) was then added to select stable cells (8–10 days). Cells were always tested for miR-101.

### Xenograft model

As previously reported^20^, eight-week-old female, nude/beige mice were purchased from Nantong University Animal Laboratories. Approximately 5 × 10^6^ 786-0 cells were injected into mice right flanks, and tumors were allowed to reach 10 mm in maximal diameter. Mice were divided into four groups (n = 10 of each group): stable 786-0 cells with scramble control shRNA, stable 786-0 cells with DNA-PKcs shRNA (-1), NU-7441 oral administration or vehicle administration. NU-7441 was initially solubilized as a stock solution of 10 mg/mL in ethanol. Prior to gavage, NU-7441 was brought up to volume (0.2 mL) in PBS with 0.5% TWEEN 80 and 2.5% *N*,*N*-dimethylacetamide. Mice body weight and bi-dimensional tumor measurements were taken every 7 days. Tumor volume was estimated using the standard formula: (length × width^2^)/2. Mice (1 mice per group) were sacrificed 7 day or 14 days after initial treatment, and the primary tumors were excised for Western blot and IHC staining analysis. Tumor xenografts were stored in liquid nitrogen. All experimental protocols were approved by the Nantong University’s Institutional Animal Care and Use Committee (IACUC, Approve ID: 2013-015) and Nantong University’s Scientific Ethical Committee (Approve ID: 2013-002). The methods were carried out in accordance with Nantong University’s IACUC regulations. Animal surgery and euthanasia using decapitation were performed under Hypnorm/Midazolam anesthesia, and all efforts were made to minimize suffering.

### Immunohistochemistry (IHC) staining

The IHC staining was performed on cryostat sections (4 μm/section) of xenograft tumors according to the described methods^32^. The slides were incubated with the primary antibody (anti-AKT Ser-473, 1:50), and subsequently stained with horseradish peroxidase (HRP)-coupled secondary antibody (Santa Cruz). The slides were then visualized via peroxidase activity using 3-amino-9-ethyl-carbazol (AEC) method (Merck, Shanghai, China).

### Statistical analyses

All experiments were repeated at least three times, and similar results were obtained. Data were expressed as mean ± standard deviation (SD). Statistical analyses were analyzed by one-way analysis of variance (ANOVA). Multiple comparisons were performed using Tukey’s honestly significant difference procedure. A ***p*** value of <0.05 was considered statistically significant.

## Additional Information

**How to cite this article**: Zheng, B. *et al.* Over-expression of DNA-PKcs in renal cell carcinoma regulates mTORC2 activation, HIF-2α expression and cell proliferation. *Sci. Rep.* **6**, 29415; doi: 10.1038/srep29415 (2016).
